# An In Vitro Study of the Abrasive Potential of Various Toothbrushes on the Surface of Aligner Attachments

**DOI:** 10.7759/cureus.55911

**Published:** 2024-03-10

**Authors:** Nazleen V Vas, Remmiya Mary Varghese

**Affiliations:** 1 Orthodontics and Dentofacial Orthopaedics, Saveetha Dental College and Hospitals, Saveetha Institute of Medical and Technical Sciences, Saveetha University, Chennai, IND

**Keywords:** resin cements, clear aligner appliances, removable, orthodontic appliances, toothbrushing

## Abstract

Background

Attachments play a vital role in aligner-led orthodontic therapy, first passively through retention of the appliance and secondly, through bringing about tooth movement, by virtue of its active surfaces, to achieve treatment goals. Additionally, irregularities on the surface of attachments attract plaque adhesion. Thus the effect of brushing with different toothbrushes on the surface of attachments is an important factor to study. This would allow clinicians to better advise patients who are undergoing aligner therapy. Four types of brushes are available commercially, namely hard, medium, soft, and ultra-soft. This study analyses the interaction between the kind of toothbrush used and the wear of the surface of the aligner attachment, to understand the impact of a toothbrush on the attachment.

Aim

To observe the surface wear and change in the shape of the aligner attachment on brushing with four varying hardness of toothbrush bristle (ultra-soft, soft, medium, and hard) over six months to three years.

Material and methods

One attachment was bonded to the buccal surface of extracted premolars. One tooth with attachment was subjected to SEM analysis and the rest were divided into four groups of five teeth each, based on the type of toothbrush to be used. Brushing with hard, medium, soft, and ultra-soft toothbrushes was carried out in a brushing simulator in two cycles simulating six months, one year, 18 months, and three years of brushing. A contact profilometer was used to evaluate surface roughness before and after brushing and pre- and post-surface roughness values were compared to quantitate changes after which SEM analysis was carried out for qualitative assessment of the surface of the samples. The Shapiro-Wilks test was applied to evaluate the normality of the data, followed by the one-way ANOVA, and statistical significance was applied at p<0.05.

Results

At six months, the samples brushed with the medium toothbrush showed the least surface roughness (0.2±0.192) and those brushed with the ultra-soft toothbrushes showed the highest surface roughness (1.9±0.159). At one year, the samples brushed with the soft toothbrush showed the least surface roughness (0.46 ±0.31) and those brushed with the ultra-soft toothbrushes showed the highest surface roughness (2.12 ±0.12). At the 1.5-year point, the surface roughness of the samples was lowest in the ultra-soft toothbrush group (0.43±0.39) and the highest in the soft toothbrush group (1.6± 0.41). At the three-year point, the surface roughness of the samples was lowest in the ultra-soft toothbrush group (0.28 ± 0.17) and the highest in the medium toothbrush group (1.6 ± 0.31).

Conclusion

Ultra-soft toothbrushes have a high abrasive potential, as seen by higher surface roughness values over six months and one year. Morphometric changes were the most noticeable for attachments brushed by hard-bristled toothbrushes and medium-bristled brushes.

## Introduction

Among the numerous types of toothbrushes available commercially, the International Organization for Standardization classifies toothbrush stiffness as soft, medium, hard, and more recently, ultra-soft based on filament stiffness [1]. Patients with erosive toothwear have been recommended to use soft-bristled toothbrushes [2]. While it would seem that brushing with soft toothbrushes is less abrasive, some in vitro studies have conversely observed that soft bristle brushes abrade the enamel surface more than those with hard bristles because greater flexion of soft bristles increases contact with the tooth surface. In addition, the higher density of tufts of soft toothbrushes would retain toothpaste on the toothbrush, thus creating a higher amount of surface contact and causing more tooth wear [3-4].

Brushing with dentifrice roughens the surface of restorations as well [5-7], and although some manufacturers lay claim to the high wear resistance of their respective composites, in vitro evaluation of such new products using simulated tooth brushing is required [8]. Studies performed to this end noted the abrasion and roughness of hybrid and nanohybrid composite resin after being brushed with toothpaste and reported that toothbrushes of varying bristle stiffness showed that surface roughness increase was directly proportional to the duration of brushing and that the stiffness of toothbrush bristles had a small effect on surface roughness post brushing [9, 10]. Orthodontic therapy with aligners requires the use of composite resin buttons or attachments on the surface of teeth. These attachments have a vital role in bringing about tooth movement, and as such their surface, shape, and form are of utmost importance for smooth and efficient treatment with aligners [10, 11]. A study of the wear of composite resin beams on brushing with three grades of stiffness of toothbrush concluded that toothbrush bristles and stiffness have a minor effect on abrasion and surface roughness of composite resins [12]. Previous studies have compared surface wear on Invisalign attachments with the use of different types of composite resin cement [12-14]. A previous study was conducted comparing surface wear of nanocomposite restorations on brushing with a soft toothbrush and medium bristle electric toothbrush. However, this study did not use a simulator to simulate tooth brushing which would introduce human error due to variation in the force of tooth brushing and pattern - linear and nonlinear - of brushing. Furthermore, evaluation of brushing over longer periods is needed to quantify the observed changes and their relation to the efficiency of orthodontic tooth movement [8].

Existing literature on this subject thus has separately explored the subjects of wear of composite resin cement as attachments and the wear of composite restorations on brushing with various stiffnesses of toothbrushes. However, no study has previously been done comparing the effects of four stiffness grades of toothbrushes on the surface of attachments placed on the buccal surface of the tooth in an environment wherein the toothbrushing parameters such as force levels, toothbrushing patterns, and brush wear have been adequately controlled. Furthermore, evidence falls short in expressing the effect of various toothbrushes on attachments over extended periods of time as is seen in clear aligner therapy. Thus the aim of this study was to observe the surface wear and change in shape of aligner attachment on brushing with four varying hardness of toothbrush bristle (ultra-soft, soft, medium, and hard) over a period of six months to three years. The primary objective is to assess the surface roughness of the aligner attachments before and after brushing with hard, medium, soft, and ultra-soft toothbrushes, in micrometers, using a contact profilometer, with an emphasis on the effect of time over the same. Secondly, the change in morphology of attachment after brushing, if any, would be visualized using a scanning electron microscope. The null hypothesis of the study states that there is no effect on the aligner attachment on brushing with a hard, medium, soft, and ultra-soft toothbrush over a period of six months, one year, 1.5 years, and three years.

## Materials and methods

In total, 21 extracted premolars were selected making sure they had no enamel fractures and caries; they were stored for a maximum of three months before use in the study. Of these, one premolar was used to prepare a control attachment that was not subjected to brushing. The 20 premolars were divided into four groups based on the duration of toothbrushing they would be subjected to. 

Attachment preparation

**Figure 1:**
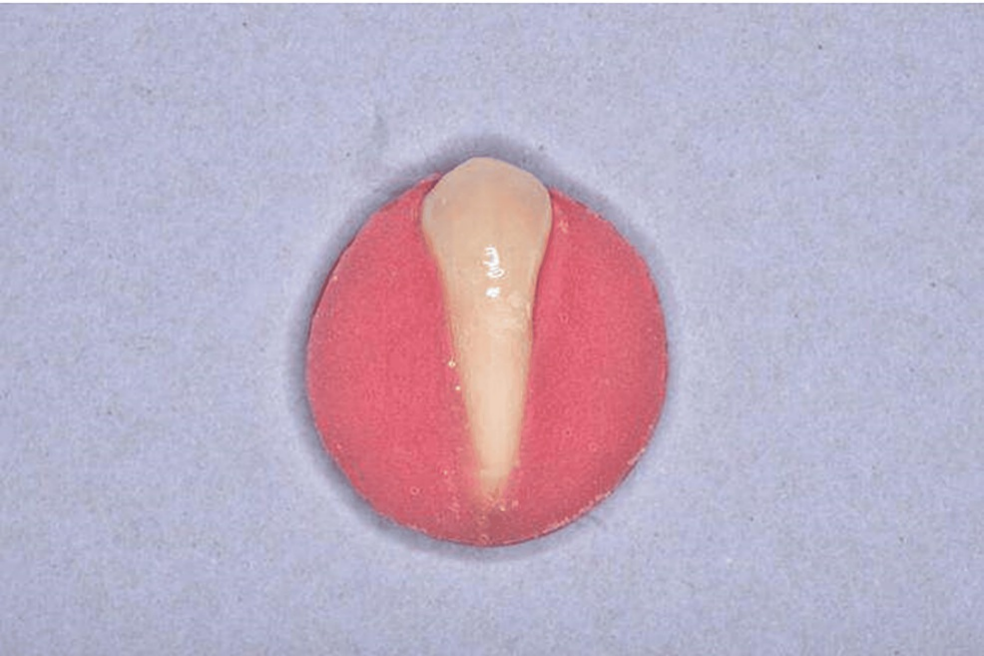
The extracted premolars were ultrasonically cleaned, dried, and mounted in resin.

Prior to attachment placement, the teeth were ultrasonically cleaned, dried, and mounted in resin (Figure 1).

**Figure 2:**
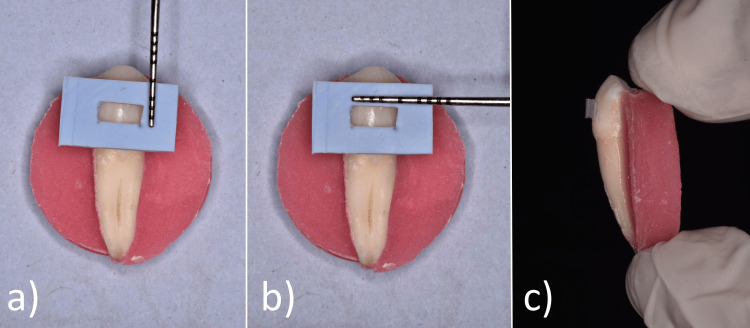
The template was fabricated such that the dimensions of the attachment was 4mm x 2mm and had a prominence of 1mm. (a) the breadth of the template was 2mm; (b)The length of the template was 4mm; (c) the depth of the template was 1mm.

A template was fabricated using putty consistency condensation silicone material, to standardize the attachment size and shape for the attachments. The thickness of the template was 1mm and a window of 4mm x 2mm was cut over the site of attachment, such that the attachment formed was 4mm wide, 2mm high, with a prominence of 1mm (Figure 2).

The enamel of the premolars was etched as per the total etch protocol using 37% phosphoric acid for 30 seconds. The acid was washed gently with water spray for 10 seconds and the enamel surface was dried for bonding agent application. 3M Filtek Transbond XT Light Cure Adhesive Primer bonding agent (3M Health Care, St. Paul, MN) was applied with a micro brush and spread thinly with compressed air.

Each attachment was placed using the template and a mylar strip and light-­cured for 10 seconds following the manufacturer’s instructions. In previous studies assessing the wear of composite materials, Filtek Z350 has shown good wear resistance [13,15], and has been recommended by Invisalign for attachment fabrication, hence this material was selected to place attachments.

The attachment surface was not polished after template removal. Baseline values for surface roughness were recorded using a contact profilometer.

Estimation of brushing time

The present study used a brushing simulator. The time taken to simulate three years of brushing through 30,00 rpm was eight hours. Accordingly, in real-time, each group was brushed for the following amounts of time: six months: 1.3 hours i.e., 78 minutes; one year: 2.6 hours i.e., 156 minutes; 1.5 years: four hours and three years: eight hours.

Depending on the severity of malocclusion, aligner therapy may last as short as 11.5 months and may go on for up to three years or more [16]. Thus considering the treatment time of the aligner for which the attachments would need to stay intact, we evaluated surface roughness at six months, one year, 1.5 years, and three years.

To account for brush wear, calculations were made as follows. It is advised to brush one’s teeth for two minutes twice a day and change one’s toothbrush every three months [17]. Applying the above conditions, it can be assumed that a toothbrush would be used for four min/day. Over a month the brush would be used for 120 minutes, and over three months the brush would be used for approximately 360 minutes or six hours. To prevent bias in the study from wear of the brush midway through the study, the brush would need to be changed every four hours.

Brushing teeth in a brushing simulator

**Figure 3:**
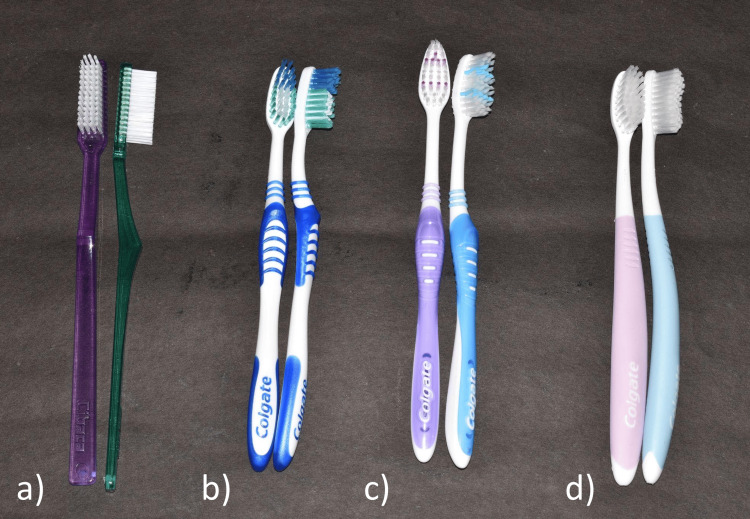
Toothbrushes used in the study (a) Colgate Cibaca Supreme Full Head (hard bristles); (b) Colgate Extra Clean Power Tip (medium bristles), (c) Colgate Super Flexi Toothbrush (soft bristles), (d) Colgate Gentle Sensitive Toothbrush (ultra-soft bristles).

The attachments were split into four groups based on the type of toothbrush to be brushed with (Figure 3). Each group had five attachments. Each group was subjected to brushing for a total of eight hours simulating three years. A common abrasive toothpaste (Colgate) was used with four types of toothbrushes from the same manufacturer for the purpose of standardization.

Specifications for Brushing Simulator

**Figure 4:**
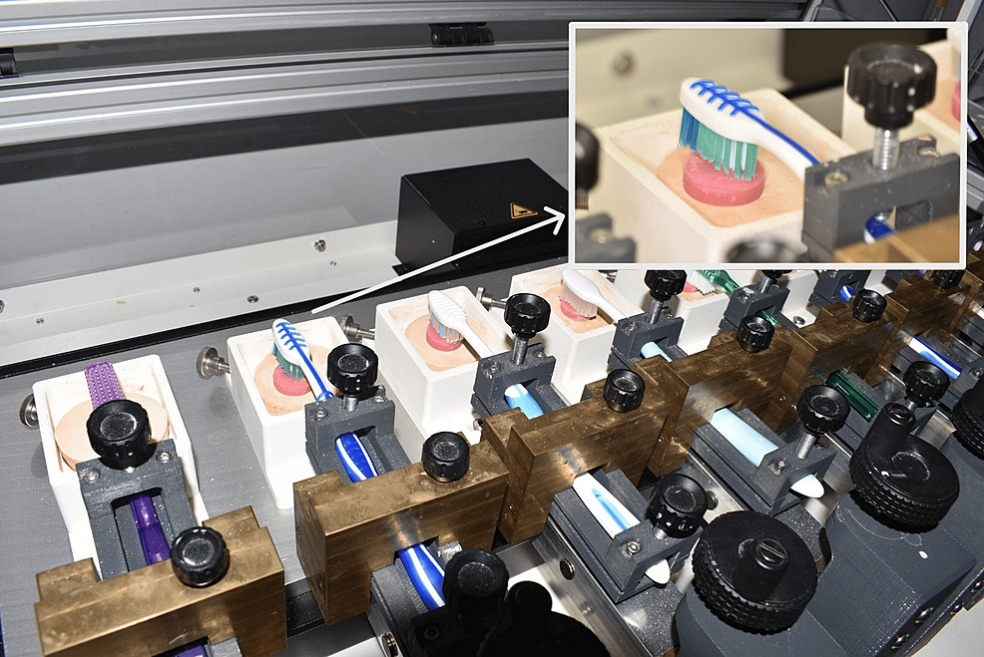
Set-up of SD Mechatronik (SD Mechatronik GmbH, Feldkirchen-Westerham, Germany) for brushing simulation with a close-up view of brush and attachment set-up in the brushing simulator

SD Mechatronik Zm3.8 (SD Mechatronik, GmbH, Feldkirchen-Westerham, Germany) was used by applying the following movement parameters to simulate the gross movement of the bristles through a combination of circular, vertical, or horizontal brushing patterns (Figure 4).

Movements incorporated were linear movement (X-axis: 10,000 cycles, Y-axis: 10,000 cycles) and circular (clockwise: 5000 cycles, anti-clockwise: 5000 cycles). Force applied on the attachments by the brushes was standardized to 2N, as is the average of adult patients.

Surface roughness evaluation by use of contact profilometry

Specifications for the Profilometer

**Figure 5:**
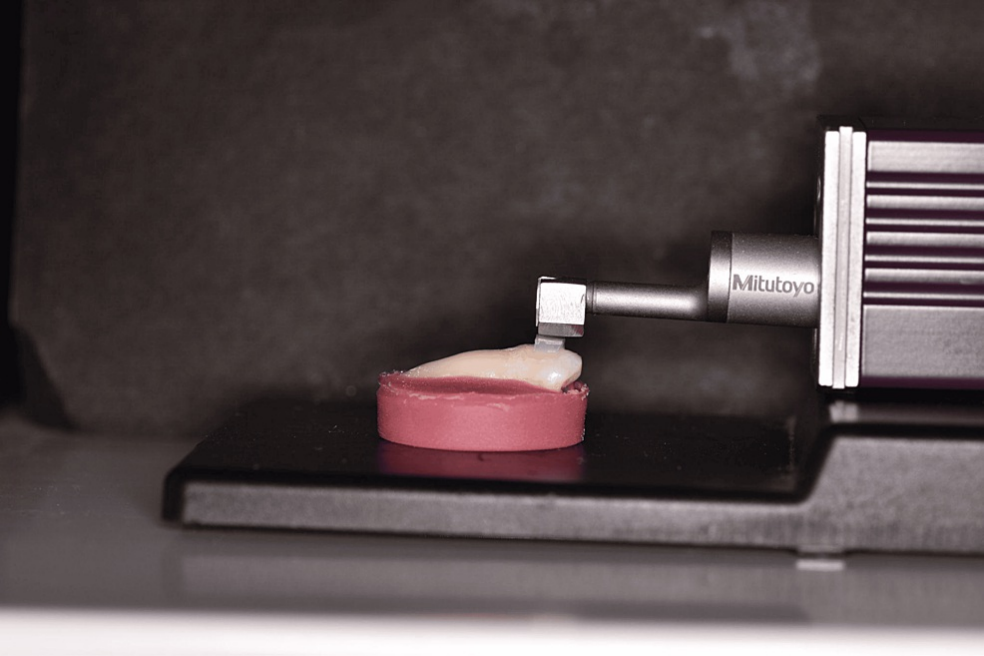
Mitutoyo SJ310 contact profilometer assessing the surface roughness of the attachment

The surface roughness of the attachments was measured using a contact profilometer before brushing, measured as t0, and after the start of the brushing simulation at the following time periods: six months (t1), one year (t2), 1.5 year (t3), three years (t4). The device used was Mitutoyo SJ310 (Mitutoyo South Asia Pvt. Ltd., Kawasaki, Japan) with a 2-micro-meter radius diamond stylus attached to the pick-up head, that runs on the surface of the attachments at a transverse speed of 0.5 mm/s at 0.75 N with automatic return (Figure 5). A calibration block was used in between attachments.

Statistical analysis was done using SPSS software v. 23 (IBM Corp., Armonk, NY). Shapiro-Wilk’s test was done to test the normality of data, and the data was found to be parametric. One-way ANOVA and Tukey's post hoc analysis were done to compare inter-group variation firstly to evaluate if there was significant statistical significance between the surface roughness of the attachments between the groups before brushing, following which a post-brushing inter-group comparison was drawn. Statistical significance was applied at p<0.05.

Specifications for scanning electronic magnification

One attachment was chosen at random from each group and as a control, was not subjected to brushing. An attachment was selected at random from the t4 subgroup of each group on completion of brushing for visual examination under a scanning electron microscope.

The attachment morphology changes post-brushing were assessed at 20 X, the change in the texture of the attachment was assessed at 50 X magnification, and a comparison was drawn to the control, on which no tooth-brushing was done. This assessment was done on a visual scale (Tables 1-2) [13].

**Table 1:** Visual Assessment Scale for Attachment Morphology

Score	Characteristic
Slight change	Roundening of the corners of the attachment
Moderate change	Roundening of the edge of the attachment along the length
Noticeable change	Facets formed over any line angle of the attachment

**Table 2:** Visual Assessment Scale for Attachment Surface Characteristics

Score	Characteristic
Slight change	Presence of diffuse porosity
Moderate change	Isolated, well-defined porosity scattered over the attachment surface with or without the presence of diffuse porosity
Noticeable change	Presence of distinct voids on the attachment surface, in addition to diffuse or scattered porosity

## Results

Evaluation of change in surface roughness of aligner attachment on brushing by virtue of contact profilometry

One-way ANOVA confirmed no statistically significant difference between the surface roughness of the attachments between the groups (Table 3).

**Table 3:** One-way ANOVA comparing the pre-brushing surface roughness of the attachments across the groups shows no statistically significant difference SD: standard deviation

Toothbrush bristle characteristics	Mean	SD	Significance
Ultra-soft brush	1.28	0.17	0.081
Soft brush	1.3	0.14	
Medium brush	1.14	0.6	
Hard brush	1.2	1.15	

Each group was studied over four simulated periods: six months, one year, 1.5 years, and three years, labelled as t1, t2, t3, and t4.

When a hard-bristled brush was used, surface roughness (Ra) values indicate that surface roughness increased after six months of brushing up to one year, following which roughness reduced in 1.5 years and further on in three years. However, surface roughness was statistically significant only in the t3 and t4 time periods. Though the medium-bristled brush group showed an increasing trend in surface roughness with time period of brushing, a statistically significant difference was seen in t1. The soft-bristled brush group shows statistically significant surface changes in surface roughness in t1. Surface roughness shows a non-linear trend, surface roughness values indicate an increase in t1, a decrease in the t2 time period an increase again in t3, and reduce in t4. The ultra-soft bristled brush group shows an increase in surface roughness in t1 and t2 following which surface roughness reduces in t3 and more so in t4. Statistically significant surface changes in surface roughness in all time periods.

Table 4 compares the difference in surface changes between the four groups at six months, one year, 1.5 years, and three years.

**Table 4:** One-way ANOVA comparing the difference in surface changes between the four groups at six months, one year, 1.5 years, and three years SD: standard deviation

Time period of brushing (Simulated)	Type of brush	Mean	SD	Significance
Six months	Ultra-soft brush	1.9	0.159	
	Soft brush	0.8	0.145	
	Medium brush	0.2	0.192	
	Hard brush	1.4	0.158	0.000
One year	Ultra-soft brush	2.12	0.12	
	Soft brush	0.46	0.31	
	Medium brush	1.02	0.31	
	Hard brush	1.54	0.24	0.000
1.5 years	Ultra-soft brush	0.43	0.39	
	Soft brush	1.6	0.41	
	Medium brush	1.4	0.47	
	Hard brush	0.74	0.18	0.000
Three years	Ultra-soft brush	0.28	0.17	
	Soft brush	0.70	0.17	
	Medium brush	1.6	0.31	
	Hard brush	0.56	0.11	0.000

**Figure 6:**
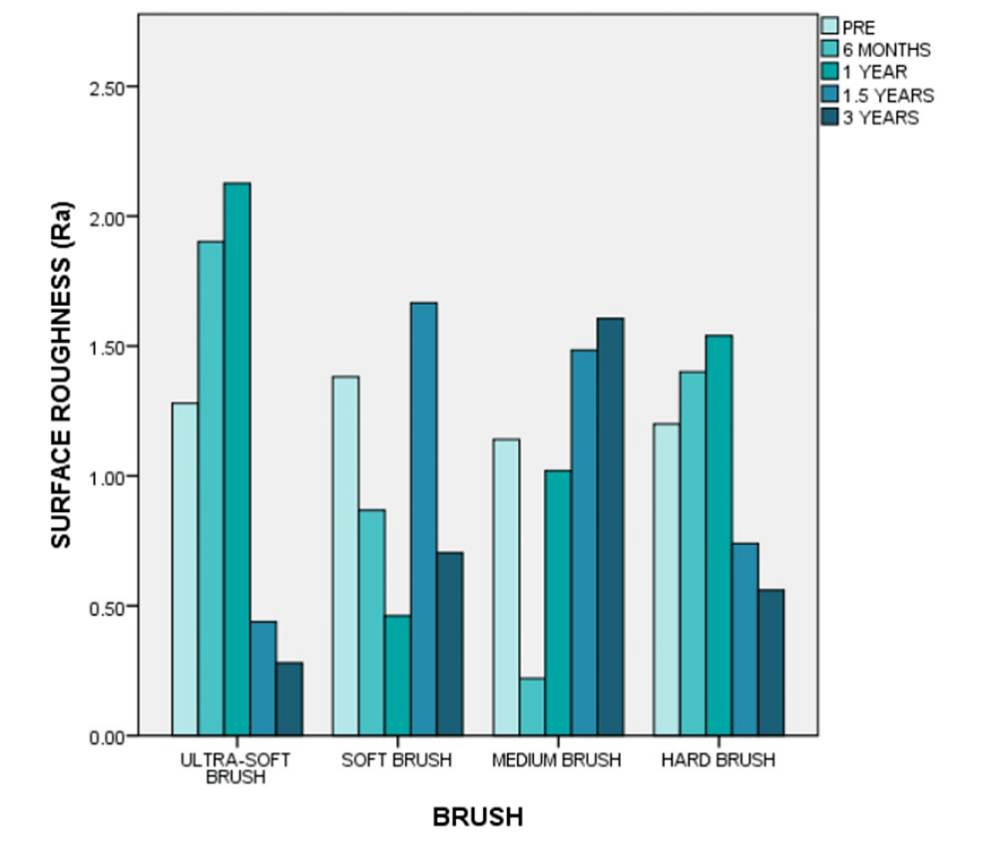
Surface roughness of the attachments on brushing with various brushes over a period of six months, one year, 1.5 years, and three years

At the six-month mark, the surface roughness of the ultra-soft brush and hard brush groups increased from their pre-brushing value, while that of the soft brush and medium brush groups reduced. After another six months of brushing, the surface roughness of the hard brush, medium brush, and ultra-soft brush groups increased while that of the soft brush reduced further. At 1.5 years of simulated brushing, the surface roughness of the ultra-soft brush group and the hard brush group reduced while that of both the other groups increased. Finally, at the three-year mark, the surface roughness of the medium brush group increased while that of the other groups reduced further. From the graph, it appears that the surface roughness of the soft brush group is closest to its pre-brushing average (Figure 6).

**Figure 7:**
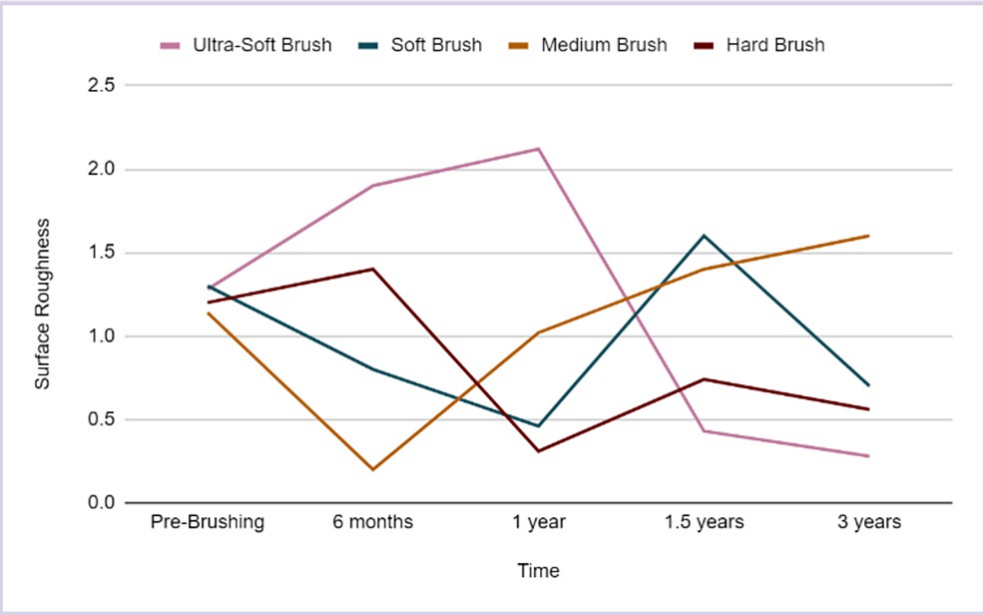
A comparison of the change in surface roughness of the attachments on brushing with various brushes over a period of six months, one year, 1.5 years, and three years.

Figure 7 illustrates the trends in surface roughness over the period of brushing. It appears that after a simulated six months of brushing, the surface roughness of the attachments increased for the group brushed with a hard and ultra-soft toothbrush and reduced for the medium and soft bristle brushes. 

SEM analysis of change in morphology and surface characteristics of aligner attachment

**Figure 8:**
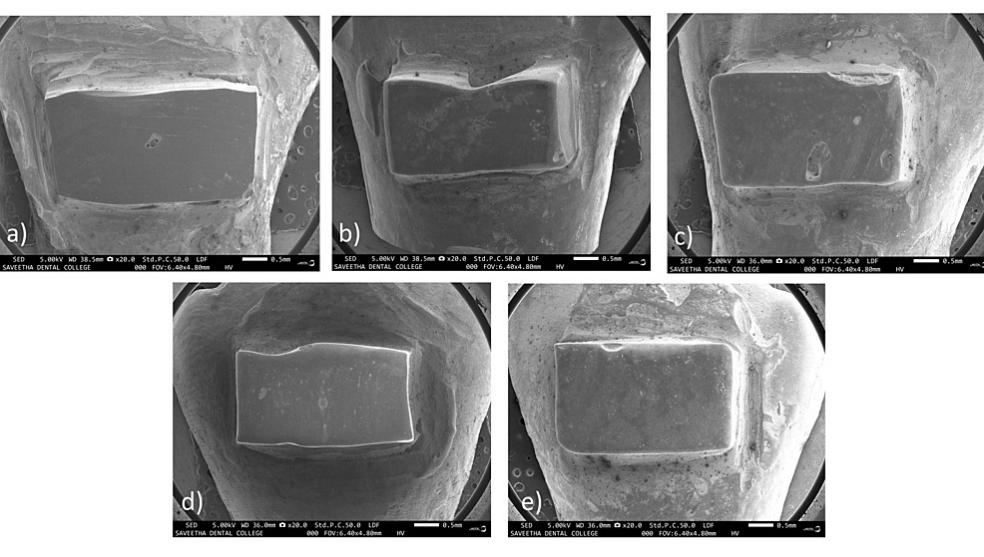
SEM images at 20X magnification of (a) unbrushed control, attachments brushed by a (b) hard-bristled toothbrush, (c) medium-bristled toothbrush, (d) soft-bristled toothbrush, and (e) an ultra-soft-bristled toothbrush SEM: scanning electron microscopy

At a magnification of 20X (Figures 8a-8e), the attachment brush by a medium brush showed the most visual change. The attachment brushed by a hard brush showed moderate change while the attachments brushed by soft and ultra-soft toothbrushes showed the least visual change (Table 5).

**Table 5:** Attachment morphology change as seen in SEM images at 20X magnification SEM: scanning electron microscopy

Brush by which the attachment was brushed	Shape		
	Slight Change	Moderate Change	Noticeable change
Hard toothbrush	-	Yes	-
Medium toothbrush	-	-	Yes
Soft toothbrush	Yes	-	-
Ultra-soft toothbrush	Yes	-	-

**Figure 9:**
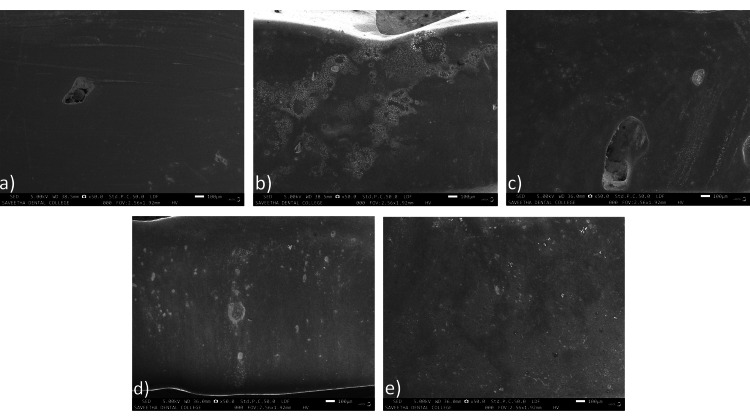
SEM images at 50X magnification of (a) unbrushed control, attachment brushed by (b) hard toothbrush, (c) medium toothbrush, (d) soft toothbrush, and (e) an ultra-soft toothbrush SEM: scanning electron microscopy

At 50X magnification (Figures 9a-9e), however, attachments brushed by both the soft and medium toothbrushes showed moderate change, while that of the hard brush showed the most change and that of the ultra-soft toothbrush showed the least change (Table 6). 

**Table 6:** Attachment texture change as seen in SEM images at 50X magnification

Brush by which the attachment was brushed	Texture		
	Slight Change	Moderate Change	Noticeable Change
Hard toothbrush	-	-	Yes
Medium toothbrush	-	Yes	-
Soft toothbrush	-	Yes	-
Ultra-soft toothbrush	Yes	-	-

## Discussion

Clear aligner therapy requires the use of auxiliary “attachments” to help reposition or retain teeth [18, 19]. A study compared complex orthodontic movements with and without attachments, showing improved efficiency of the appliance with attachments. Aligner treatment uses attachments to achieve better predictability of orthodontic tooth movement [11, 20]. Studies in vitro and in vivo noted wear of composite resins on contacting the occlusal surface of the antagonist tooth in Class I restorations and by the mechanical action of brushing, diet, and salivary pH [8, 21, 22]. Surfaces of composite resin restorations may be negatively impacted by a range of oral environment variables. The labial and buccal surface of the teeth and attachments however would be largely impacted by daily hygiene practices like toothbrushing [23]. Therefore, it was vital to conduct this investigation to determine how brushing affected the surface integrity of aligner attachments.

The experimental conditions of the present investigation were designed to simulate the intraoral effect of brush and toothpaste on aligner attachments. A brushing simulator was used to ascertain that the same amount of pressure and brushing pattern was applied across all the groups, and a brush change was included to account for brush wear and replacement after three months of brushing. In agreement with the load range specified in ISO/TR 14569-1, the force applied by the brush against the specimen was calibrated to 2 N. For the determination of the maximum depth of wear, the graphical determination of wear depth from profilometer tracing, as used in the present investigation is a common and suitable procedure [6]. It is necessary to reject the null hypothesis, which claimed that the grade (stiffness) of the toothbrush employed had no impact on the wear, texture, or roughness of the composite resin.

A study done by Zairani et al. [7] had previously evaluated the surface roughness of nanocomposite (Filtek Z 250 XT, 3M ESPE) material over three months, comparing the use of medium stiffness and soft bristle electric toothbrushes, concluding that soft brushes showed no statistically significant surface roughness, however, medium stiffness brush showed an increase in surface roughness after a simulated two months of brushing. The shortest time period considered in our study was six months. Our study also compared two more brush stiffness, i.e. hard brush, and an ultra-soft toothbrush, and the results indicated that ultra-soft bristles caused the most abrasion, followed by a hard and medium toothbrush. Soft brushes caused the least surface roughness. 

In other studies by Souza et al. [8] and Gomez et al. [10], the surface roughness of various types of composite material tested with hard, medium, and soft brushes was studied over a simulated period of one year. Two of the composite cement types used had a filler volume percentage of 59% and 64%. The composite cement used in our study has a filler volume of 60% and the results of both studies may be compared. The composite resin cement with 59% filler volume showed more surface roughness on brushing with soft and medium brushes. The composite resin with a volume percentage of 64% showed no statistically significant surface roughness. In our study, on one year of brushing, a hard toothbrush showed the most surface roughness, followed by a medium stiffness toothbrush and then a soft toothbrush. SEM images showed surface differences such as reduction and/or presence of porosities on the surface of the attachment, and increased surface irregularities in comparison with a control that was not subjected to brushing. Comparison of initial and final images showed that all attachments underwent some degree of modification of the surface texture, but there was never total destruction of the attachment and the change in surface morphology is not clinically significant. Based on the results of our study, it appears that soft-bristled brushes produce the least amount of change in the morphology of aligner attachments, and ultra-soft toothbrushes produce the least change on the attachment surface at a microscopic level. Hard bristled brushes are deleterious to the health of gingiva and are conduit to wear of enamel [10, 24, 25].

Limitations

The limitation of the study is that despite the best efforts to reproduce the wear of aligner attachments in patients in an in vitro study of the same, an in vivo study would be in order to assess the wear of attachments between left-handed and right-handed patients and to account for variation in brushing patterns, force and time across various demographics. This study evaluated the abrasive potential of toothbrushes as a function of the surface wear of the attachments, however, a cross-sectional examination of the attachments after brushing to evaluate volume loss since the change to the shape of the attachment may impair its function. In addition, the wear of the attachment could have been better assessed by viewing the cross-section of the attachments. Another parameter worth exploring would be if end-rounded bristle tips would be less likely to cause attachment abrasion.

## Conclusions

Considering the results of our study, we may conclude that soft-bristled brushes cause the least surface wear of the aligner attachment as compared to hard- and medium-bristled brushes. Ultra-soft toothbrushes produce the least change on the attachment surface at a microscopic level, while soft-bristled brushes are a safer choice in oral hygiene maintenance, keeping in view gingival health and lower rates of enamel surface wear. The interplay of the effect of the bristle with the effect of abrasive in the dentifrice is vital in understanding the effect of brushing on teeth and composite resin attachments. Future research should include in vivo and longitudinal studies to clarify the relationship between the degree of end-rounding of toothbrush bristles as well as that of brush bristle stiffness to the wear of aligner attachments.
